# *Aspergillus* Infections in Cetaceans: A Systematic Review of Clinical, Ecological, and Conservation Perspectives

**DOI:** 10.3390/biology14060664

**Published:** 2025-06-07

**Authors:** Victor Garcia-Bustos, Inmaculada Rosario Medina, Marta Dafne Cabanero-Navalon, Rosie S. Williams, Shaheed Karl Macgregor, Shinto Kunjamma John, Francisco Javier Aznar, Patricia Gozalbes, Begoña Acosta-Hernández

**Affiliations:** 1 Severe Infection Research Group, Health Research Institute La Fe, 46026 Valencia, Spain; cabanero_marnav@gva.es; 2 Instituto Universitario de Sanidad Animal y Seguridad Alimentaria (IUSA), Universidad de las Palmas de Gran Canaria, 35413 Arucas, Spain; bego.acosta@ulpgc.es; 3 Institute of Zoology, Zoological Society of London, London NW1 4RY, UK; rosie.williams@ioz.ac.uk (R.S.W.); shaheed.macgregor@zsl.org (S.K.M.); shintokunjamma.john@ioz.ac.uk (S.K.J.); 4 Marine Zoology Unit, Cavanilles Institute of Biodiversity and Evolutionary Biology, Science Park, University of Valencia, 46010 Valencia, Spain; francisco.aznar@uv.es (F.J.A.); patricia.gozalbes@uv.es (P.G.)

**Keywords:** *Aspergillus*, cetaceans, marine mammals, aspergillosis, antifungal resistance, zoonotic fungi, One Health, climate change, conservation medicine, morbillivirus coinfection

## Abstract

*Aspergillus* spp., particularly *A. fumigatus*, are increasingly reported as emerging pathogens in cetaceans, yet their clinical and ecological relevance remains poorly characterized. This systematic review synthesizes evidence from 34 studies involving 106 animals, identifying respiratory, neurological, and otic infections as the most frequent presentations with potential interspecies tropism. Invasive disease, frequently fatal, was linked to co-infections—especially with morbillivirus—and environmental stressors such as pollution- and climate-related immune suppression. Despite cetaceans’ role as sentinel species, antifungal susceptibility testing and species-level identification were inconsistently performed. Additionally, azole-resistant *A. fumigatus* strains were isolated from wild porpoises, indicating environmental antifungal exposure and potential public health implications. Aspergillosis remains underdiagnosed in free-ranging populations, particularly in remote or pelagic species. Conservation implications were scarcely addressed, despite evidence suggesting that fungal disease may contribute to morbidity, stranding, and population impact. This review underscores the need for enhanced surveillance, integrative diagnostics, and recognition of fungal pathogens in a One Health framework. The growing intersection of climate change, emerging mycoses, and wildlife conservation positions *Aspergillus* infections in cetaceans as both a marine mammal health concern and an ecological indicator of broader environmental changes.

## Simple Summary

Fungal infections caused by *Aspergillus* species, especially *Aspergillus fumigatus*, are being increasingly detected in whales, dolphins, and porpoises. These infections can affect the lungs, brain, and ears of the animals, and in many cases lead to serious illness or death. Despite their importance, fungal diseases in cetaceans are often overlooked in both research and conservation efforts. This study reviews all available published cases of *Aspergillus* infections in these animals to understand how and where they occur, how they are diagnosed, and what treatments have been used. We found that most infections were found in stranded and captive cetaceans, which are easier to study. Many animals were also infected with other infective agents, especially viruses, suggesting that their immune system was impaired. Infections were more common in areas affected by pollution or climate change. Alarmingly, fungal sensitivity to antifungal medicines was barely tested, even though resistance is a growing concern for both animal and human health. Our findings highlight the urgent need for better disease monitoring, earlier diagnosis, and more attention to fungal infections in cetaceans. These infections may be sentinels of larger environmental problems that affect both wildlife and people.

## 1. Introduction

Cetaceans play a crucial role in marine ecosystems by regulating predator–prey dynamics and contributing to nutrient cycling across trophic levels [1,2,3]. Their high trophic position, long lifespan, and capacity to bioaccumulate toxins make them essential sentinel species for both oceanic and human health [4,5,6]. However, these species are increasingly exposed to severe environmental threats, including climate change, pollution, and emerging infectious diseases, which are often interlinked and may significantly impact their populations and, consequently, marine biodiversity [6,7]. These stressors often act synergistically: climate change may alter pathogen dynamics and weaken host immunity, while pollutants can exacerbate susceptibility to infection—particularly in inshore species, which are more heavily exposed to further anthropogenic pressures [6].

Both captive and wild cetaceans are susceptible to a range of infectious diseases caused by bacteria, viruses, fungi, and parasites. Notably, pathogens, including cetacean morbilliviruses, herpesviruses, *Brucella* spp., or *Toxoplasma gondii* (Nicolle & Manceaux, 1908), among others, can disrupt population dynamics by increasing mortality, reducing reproductive success, or exacerbating the severity of co-infections [6,8]. While bacterial and viral infections in marine mammals are well-studied, fungal diseases remain underexplored, despite the growing recognition, particularly in the context of climate change, that they may contribute to the emergence and increasing impact on cetaceans, other marine animals, and humans [8,9,10,11,12].

Fungal infections in cetaceans have been primarily linked to *Paracoccidioides ceti* (Vilela, Mendoza, Rosa, Giannini, Larsson & Vilela) (formerly *Lacazia loboi* Taborda, V.A. Taborda & McGinnis) with mainly cutaneous involvement, or *Aspergillus* spp., opportunistic yeasts such as *Candida* spp., and Mucorales, among others, which cause systemic disease with respiratory or neurological affectation [10,11,13,14,15,16,17,18]. Despite these known associations, knowledge about the prevalence, pathogenicity, transmission dynamics, and conservation implications of these fungal pathogens remains limited. Given the emergence of fungal pathogens in marine environments, the role of fungi in cetacean diseases is expected to become more significant in the future [19]. The rising incidence of fungal infections has been directly linked to broader ecosystem disturbances, including pollution and climate variability, which can influence pathogen dynamics and increase the susceptibility of hosts [4,5,20]. Despite the recognized ecological significance of marine mammals, fungal-associated diseases are rarely integrated into conservation strategies, highlighting a critical gap in disease surveillance.

Beyond their conservation implications, cetaceans may also serve as reservoirs for zoonotic fungal pathogens, raising concerns about public health risks [20]. Human–cetacean contact may occur during cetacean shows, ecotourism activities, and occupational exposure related to handling live animals or cetacean carcasses. These interactions pose direct risks to professionals such as veterinarians, researchers, trainers, and fisheries workers, who may experience prolonged exposure to cetaceans and their biological fluids [21,22,23]. Occupational exposure during stranding events, particularly when handling infected carcasses, represents a potential route for fungal zoonotic transmission, which may be exacerbated by inadequate protective measures in fisheries settings [23]. This issue not only poses threats to human health but also has economic consequences, since contamination risks could impact seafood market safety and consumer confidence [23].

*Aspergillus* spp., particularly *Aspergillus fumigatus* Fresen, are increasingly implicated in respiratory, neurological, and systemic infections across multiple cetacean species, contributing to morbidity, mortality, and potential population declines [8,24,25,26,27]. These infections threaten both wild and captive populations, highlighting a growing conservation concern; however, most evidence is limited to isolated reports [24,28]. The rise of environmentally acquired azole-resistant *A. fumigatus*, driven by antifungal overuse in livestock and agriculture as well as contamination into marine ecosystems, further exacerbates this threat. It compromises treatment efficacy in animals and humans, and underscores the interconnected risks between marine wildlife and the health of domestic animals and humans, particularly for professionals handling stranded or bycaught cetaceans [29,30,31,32,33]. Given the role of cetaceans as sentinel species, the emergence of *Aspergillus* spp. as a conservation challenge reflects broader environmental disturbances that need to be addressed.

In this study we provide a systematic review of *Aspergillus* infections in cetaceans assessing their clinical, ecological, and conservation significance.

## 2. Materials and Methods

### 2.1. Data Search

This review conforms to the PRISMA (Preferred Reporting Items for Systematic Reviews and Meta-Analyses) guidelines [34]. The PRISMA checklist has been included in Supplementary Material. To precisely define the research question and streamline the literature review, the PICO framework (Population, Intervention, Comparison, Outcome) was applied [35]: (i) population: cetaceans; intervention: *Aspergillus* species; comparison: not applicable for this review; outcome: documented evidence of *Aspergillus* species infection or colonization. Relevant studies were retrieved from the databases PubMed MEDLINE, Web of Science, Scopus, and Google Scholar using the following Boolean query: (“aspergillus” OR “aspergillosis”) AND (“cetacean” OR “whale” OR “dolphin” OR “porpoise” OR “mysticete” OR “mysticeti” OR “odontocete” OR “odontoceti”). The systematic literature review was conducted from January 2025 to March 2025. The date of the last search was 10 March 2025. No public registration of the protocol was performed.

### 2.2. Study Selection and Endpoint

A systematic selection process was implemented to ensure methodological validity. Studies were eligible if they provided microbiological, histopathological, or molecular confirmation of *Aspergillus* colonization or infection in free-living cetaceans or those under human care. Only primary research articles, including case reports, case series, and observational studies, were considered. To ensure clinical relevance, studies needed to report data on clinical presentation, microbiology, pathology, epidemiology, coinfections, antifungal susceptibility, or treatment outcomes. Exclusion criteria included the absence of primary data (e.g., reviews or editorials), lack of relevance to cetaceans or *Aspergillus*, duplication, insufficient diagnostic confirmation, exclusive focus on experimental infections, or publication in languages other than those specified without accessible data. To enhance completeness, the reference lists of included studies were manually reviewed to identify additional relevant literature.

The study endpoint was to characterize *Aspergillus* spp. infections and colonization in cetaceans, including the type of infection, affected species, microbiological identification methods, antifungal susceptibility, treatment approaches, and clinical outcomes.

### 2.3. Data Extractions and Definitions

Data from each eligible study were independently extracted by two investigators (VGB, MDCN). Extracted variables included study characteristics (type of study, year of publication, and location), cetacean species (including free-ranging or captivity status), and number of affected individuals. According to their location and habitat, cases were assigned a simplified climate class based on Köppen–Geiger climate regions [36] and grouped into tropical/temperate (A + C) vs. continental/polar (D + E) to explore temperature relations and *Aspergillus* detection. Microbiological data comprised the reported *Aspergillus* species, the diagnostic method(s) used (e.g., culture, histopathology, molecular identification), and coinfections (including co-isolated pathogens). Clinical variables included clinical diagnosis (e.g., tracheitis, bronchopneumonia, encephalitis), or colonization, antifungal susceptibility testing, including its methodology and results, and the treatment administered (if reported). The primary outcome was the clinical course, categorized as resolution or attributable death according to the study. When available, the relationship between *Aspergillus* infection and attributable death was recorded according to the study authors. Additionally, when available, we extracted information on potential conservation implications and ecological context relevant to *Aspergillus* infections. Any discrepancies between the two reviewers were resolved by consensus.

### 2.4. Statistical Analysis

Statistical analyses were performed using R version 4.4.1 (RStudio 2024.12.1+653). Descriptive statistics were calculated for quantitative and categorical variables. Fisher’s exact test was applied to categorical comparisons when pertinent, and a two-sided *p*-value < 0.05 was considered statistically significant.

## 3. Results

### 3.1. Literature Search

**Figure 1:**
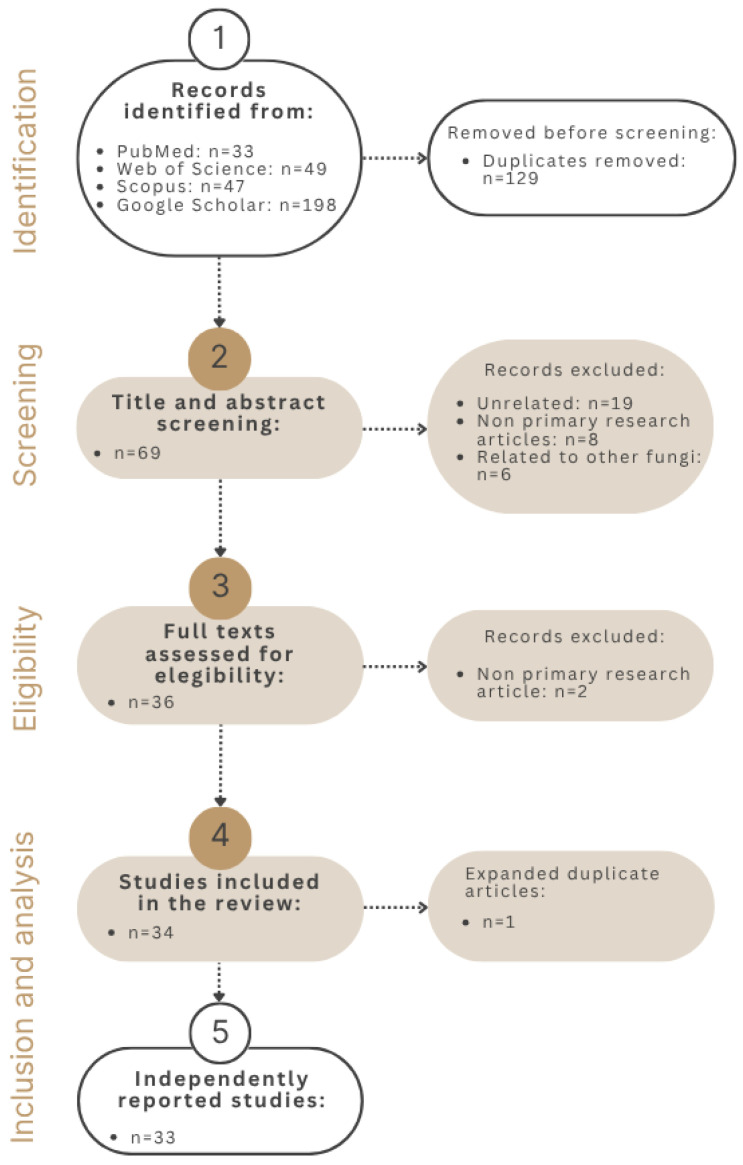
PRISMA flow diagram of literature search, eligibility, and inclusion process.

A total of 327 articles were identified, among which 198 were retrieved through Google Scholar, 33 from PubMed, 49 from Web of Science (WoS), and 47 from Scopus. After removing 129 duplicate records, 69 articles remained for title and abstract screening. Following it, 33 articles were excluded: 19 were unrelated to the research topic, 8 were non-primary research articles, and 6 focused on other fungal species rather than the target taxa. A total of 36 articles were then selected for full-text review. Of these, two articles were excluded because they were non-primary research articles. Ultimately, 34 studies met the inclusion criteria for the review. One study was excluded as a duplicate with expansion, as it reported the same case with additional information, but all relevant information on the case in both publications was jointly incorporated in the same entry [37,38]. Thus, a total of 33 independently reported studies were included in the final review. The process is graphically represented in Figure 1.

### 3.2. Features of Selected Studies

Of the 33 selected studies, 8 (23.5%) were published before 2000 and 26 (76.5%) in 2000 or later. Case reports predominated (*n* = 16, 48.5%), followed by case series (*n* = 4, 12.1%), and various observational designs: epidemiological (*n* = 6, 18.2%), ecological and microbiological (*n* = 1, 3.0%), microbiological (*n* = 1, 3.0%), and retrospective observational (*n* = 1, 3.0%). We also found one retrospective cross-sectional microbiological study (*n* = 1, 3.0%), one environmental surveillance study (*n* = 1, 3.0%), one experimental research article (*n* = 1, 3.0%), and one technical note (*n* = 1, 3.0%). Most of the surveys (27, 81.8%) dealt with fungal or Aspergillus infections, while 6 (18.2%) did not focus on fungi and the reported findings were incidental. Further information is presented in Table 1.

### 3.3. Reported Cases and Affected Cetacean Species

The total number of cetacean individuals reported in the 33 studies was 106, of which 19 (17.9%) were included in publications before 2000, and 87 (82.1%) in publications from 2000 or later. Sixteen cetacean species were involved, with bottlenose dolphins *Tursiops truncatus* Montagu, 1821 being the most frequently reported species to be infected or colonized by *Aspergillus* spp. (13 studies [39.4%] and 51 animals [51.5%]). Harbour porpoises *Phocoena phocoena* L. were reported in six studies (18.2%) and 21 affected individuals (21.2%), whereas killer whales *Orcinus orca* (L.), Atlantic spotted dolphins *Stenella frontalis* (Cuvier, 1829), and striped dolphins *Stenella coeruleoalba* (Meyen, 1833) were each reported in two studies (6.1%), with 1, 2, and 4 affected individuals, respectively (Table 1).

For the other 11 cetacean species, single studies were found, all involving single animals, except for the beluga *Delphinapterus leucas* (Pallas, 1776) and the false killer whale *Pseudorca crassidens* Owen 1846, with nine and six cases, respectively (Table 1). Overall, *Aspergillus* ssp. were detected in one mysticete species and a wide array of odontocetes from six families (Table 1).

The chronological distribution of *Aspergillus* infections and colonization evidence in cetaceans varied across the reported studies. Documented cases of *Aspergillus* infection or colonization in cetaceans were scarce before 2000, with only six studies (18.18%) from the 1990s and five (15.15%) from the 2000s. Most reports were published from 2010 onward. However, eight studies (24.24%) lacked sufficient temporal information to determine when infections occurred (Table 1).

Of the reported cases, 21 studies (63.6%) documented infections in wild, free-living cetaceans, while 10 studies (30.3%) described cases in animals under human care. Two studies (6.1%) included both captive and wild cetaceans. Among animals under human care, *T. truncatus* was the most frequently reported species (42 animals, 80.8%), followed by *D. leucas* (9, 17.3%) and one *O. orca* (1.9%). One study also isolated *Aspergillus* spp. from an aquarium pool environment linked to two cases in killer whales, though it did not assess other animals [28].

In free-ranging cetaceans, *P. phocoena* was the most commonly affected species (21 cases, 38.2%), followed by *T. truncatus* (10, 18.2%) and *P. crassidens* (6, 10.9%). *T. aduncus* and *S. coeruleoalba* each accounted for four cases (7.3%).

The geographical distribution of reported *Aspergillus* infections in cetaceans was analysed across large-scale regions. North America and Europe had the highest number of reports, with nine studies each (27.3%) documenting cases in the USA, Canada, the UK, the Netherlands, Germany, Spain, and Italy. South America accounted for three studies (9.1%), with cases reported in Brazil, Argentina, and Chile. Australia contributed two reports (6.1%), while Canada contributed a single one (3.0%) (Table 1).

### 3.4. Aspergillus Species and Identification Approaches

Among the included studies, 13 reports (39.4%) did not reach species-level identification, referring to isolates as *Aspergillus* spp. or *Aspergillus*-like structures. In total, seven *Aspergillus* species were identified. The most frequently reported species was *A. fumigatus*, which appeared in 19 studies (57.6%), either alone or in combination with other *Aspergillus* species. Other identified species included *A. niger* Tiegh. (four studies, 12.1%), *A. versicolor* (Vuill.) Tirab. (two studies, 6.1%), and *A. terreus* Thom (two studies, 6.1%). Additionally, *A. ustus* (Bainier) Thom & Church, *A. tubingensis* Mosseray, and *A. flavus* Link were each reported in a single study (3.1%) (Table 2).

With regard to the number of hosts involved, *A. fumigatus* was detected in 42 animals (77.8%), of which 35 (83.3%) were associated with invasive infections and 7 (16.7%) were identified as respiratory colonization without evidence of invasive disease. *Aspergillus niger* was reported in five animals (9.3%), with four cases of colonization (80.0%) and one invasive case (20.0%). *Aspergillus terreus* was isolated in three animals (5.6%), all of which were invasive infections, whereas *A. flavus* was found in two animals (3.7%), exclusively in cases of respiratory colonization. *Aspergillus versicolor*, *A. ustus*, and *A. tubingensis* were each identified in single invasive cases. *Aspergillus fumigatus, A. niger, A. versicolor* were also found in a study of the fungal microbiota of the aquarium environment of killer whales kept in captivity (Table 2).

A total of 106 animals were included in the climatic distribution analysis. Of these, 72 (67.9%) were reported from tropical or temperate regions (Köppen-Geiger classes A—4 studies—and C—19 studies), while 34 (32.1%) originated from continental or polar climates (classes D—8 studies—and E—2 studies). Most cases occurred in temperate zones, particularly in one study reporting 32 animals. Only five cases were associated with polar climates, and no cases from tropical regions involved more than one animal.

Various identification techniques were used to diagnose *Aspergillus* infections in cetaceans (Table 2). Culture-based methods were the most frequently applied, reported in 17 studies (51.5%), sometimes in combination with additional methods such as DNA sequencing (4 studies, 12.1%), histopathology (3 studies, 9.1%), PCR (2 studies, 6.1%), serology (1 study, 3.0%), and galactomannan detection (1 study, 3.0%). Histopathology alone was performed in six studies (18.2%), while an additional four studies (12.1%) combined histopathology with PCR or DNA sequencing. A comprehensive approach incorporating multiple methods, including culture, histopathology, PCR, and DNA sequencing, was applied in three studies (9.1%), ensuring a more robust identification. However, three studies (9.1%) did not provide specific details on the identification techniques used.

Antifungal susceptibility testing was performed in 2 out of 33 studies (6.1%), while 30 studies (90.9%) did not conduct susceptibility testing. One study (3.0%) did not provide data regarding antifungal susceptibility. The methodologies used included the EUCAST broth microdilution reference method and VIPcheck™ azole resistance screening. Among the 33 analysed studies, only 2 (6.1%) performed antifungal susceptibility testing, both using the EUCAST broth microdilution reference method. One study in *T. truncatus* reported that *A. fumigatus* exhibited high-level resistance to itraconazole and voriconazole (MIC > 16 mg/L) and low-level resistance to posaconazole (0.5 mg/L), while remaining susceptible to amphotericin B (MIC 1 mg/L) (see Table 2).

The second study further explored azole resistance using VIPcheck™ screening, identifying two out of six *A. fumigatus* isolates from *P. phocoena* as resistant. One isolate carried the TR34/L98H mutation, conferring resistance to itraconazole (>16 mg/L), voriconazole (2 mg/L), posaconazole (0.25 mg/L), isavuconazole (2 mg/L), and miconazole (>16 mg/L), while remaining susceptible to amphotericin B (0.25 mg/L). The second isolate harboured the C(-70)T/F46Y/C(intron7)T/C(intron66)T/M172V/E427K mutations, showing a similar azole resistance profile, with higher voriconazole (4 mg/L) and posaconazole (1 mg/L) MICs, while amphotericin B remained effective (0.5 mg/L) (Table 2).

### 3.5. Infection Types and Management

Among the 32 studies that reported *Aspergillus* in cetaceans (1 study only examined the facilities where animals were kept), lower respiratory infections were the most prevalent, being documented in 21 studies (65.63%). These included pneumonia, bronchopneumonia, necrotizing pneumonia, and lung abscesses, often characterized by angioinvasion, granulomatous inflammation, and extensive tissue necrosis. *Tursiops truncatus* was the most frequently affected species (five studies), followed by *P. phocoena* (four studies). Other affected species included *S. coeruleoalba*, *H. ampullatus*, and *P. blainvillei*, each with one study reporting pulmonary disease.

Upper respiratory tract infections were described in eight studies (25.0%), including tracheitis, tracheobronchitis, and sinusitis, frequently occurring alongside lower respiratory pathology. *Tursiops truncatus* was again the most frequently affected species (three studies), followed by *S. coeruleoalba* and *S. frontalis* (one study each). In several cases, occlusive tracheitis with fibrinous exudates was observed, leading to airway obstruction.

Neurological involvement was reported in seven studies (21.9%), with diagnoses of encephalitis, meningoencephalitis, and cerebral necrosis. *Phocoena phocoena* was the species most frequently affected, with three studies documenting central nervous system involvement, followed by *S. coeruleoalba* and *H. ampullatus*, each with one study. *Tursiops truncatus* was reported to have neurological disease in one study, usually occurring in conjunction with respiratory infection. Pathological features included multifocal granulomas, necrotizing vasculitis, and haemorrhagic lesions in the brain and meninges. Otitis media or interna was reported in three studies (9.4%), all involving *P. phocoena*, characterized by suppurative inflammation, tympanic osteolysis, and fungal hyphae infiltration of the middle ear structures. No other species were reported with fungal ear infections.

Additional manifestations included cutaneous infection (one study, 3.2%), observed in *Pseudorca crassidens*, and systemic or disseminated infections (five studies, 15.6%), which frequently involved multiorgan necrotizing granulomatous lesions affecting the lungs, brain, lymph nodes, myocardium, or gastrointestinal tract. These findings document the distribution of *Aspergillus* infections across cetacean species, with *T. truncatus* being the most frequently affected (14 studies, 43.8%), followed by *P. phocoena* (6 studies, 18.8%) (see Table 3).

Among the 32 analysed studies, 19 (59.4%) reported co-infections, 10 (31.3%) explicitly stated the absence of co-infections, and 3 studies (9.4%) did not clarify whether other infectious agents were found (Table 3). The most frequently documented co-infecting agent was morbillivirus, identified in five studies (26.3% of co-infection cases). Other bacterial co-infections included *Pseudomonas aeruginosa* (Schroeter, 1872) Migula, 1900; *Escherichia coli* (Migula, 1895) Castellani & Chalmers, 1919; *Streptococcus pyogenes* Rosenbach, 1884; *Staphylococcus aureus* Rosenbach, 1884; *Fusobacterium* sp., *Morganella morganii* (Winslow et al., 1919) Fulton, 1943; and *Photobacterium damselae* (Love et al., 1981) Brenner et al., 1986, among others. Fungal co-infections were less commonly reported, but *Fusarium equiseti* (Corda) Sacc., and *A. terreus* in a coinfection with *A. tubingensis* in *Pseudorca crssidens* were isolated alongside other pathogens. Parasitic and viral co-infections included *Sarcocystis speeri* Dubey & Lindsay, 1999, *Treponema* sp., *Pseudalius inflexus* (Diesing, 1851), *Stenurus minor* (Kuhn, 1829), Alphaherpesvirus, and *Mucorales*. No significant differences were seen in the prevalence of coinfections in wild vs. captive animals (Fisher’s test, *p* = 0.181).

Regarding management, despite a significant amount of records reporting stranded cetaceans, some studies on animals under human care describe management strategies and outcomes of *Aspergillus* infections. Among the 33 studies analysed, antifungal treatment was reported in 4 studies dealing with animals under human care. The most frequently administered antifungal was itraconazole, used in two studies (6.3%). Other reported treatments included fluconazole (one study, 3.1%), voriconazole in combination with posaconazole (one study, 3.1%), and a combined regimen of voriconazole, micafungin, and inhaled amphotericin B (one study, 3.1%).

Among the 32 studies analysed, fatal outcomes were reported in 20 studies (62.5%). Four studies (12.5%) documented successful resolution following antifungal treatment, while two studies (6.3%) described animals in good health despite fungal colonization or infection. One study (3.1%) reported non-severe skin lesions as the only attributable outcome, without reporting treatment because free-ranging cetaceans (*P. crassidens*) were involved. The outcome was uncertain in one case (3.1%), where the animal died due to bycatch, making it unclear whether *Aspergillus* infection contributed to mortality. Four studies (12.5%) did not specify the outcome.

### 3.6. Implications for Conservation

Of the 33 studies analysed, 19 (57.6%) did not discuss conservation implications related to *Aspergillus* infections in cetaceans. Eighteen studies (39.4%) provided partial references, typically in the context of co-infections, environmental stressors, or immunosuppression, but without explicitly linking fungal infections to conservation concerns. Only three studies (9.1%) directly addressed the conservation significance of *Aspergillus* infections, emphasizing the role of disease surveillance in marine mammal health monitoring, the potential impact of immune suppression due to environmental contaminants, and the need to consider fungal infections in wild cetacean populations.

Notably, four studies (12.1%) reported morbillivirus as a predisposing factor for invasive aspergillosis, suggesting that viral–fungal co-infections may exacerbate mortality risks. Five studies (15.2%) highlighted the potential role of environmental pollutants in immunosuppression, which could increase susceptibility to fungal infections. Three studies (9.1%) suggested that climate change and anthropogenic stressors may contribute to the rising prevalence of fungal infections in marine mammals, particularly in species inhabiting heavily impacted coastal environments. Two studies (6.1%) discussed the importance of cetaceans as sentinel species, advocating for systematic pathological investigations of stranded individuals to monitor fungal disease trends. Additionally, one study (3.1%) noted that fungal infections might impair echolocation, potentially affecting foraging efficiency and increasing stranding risk.

## 4. Discussion

This review synthesizes the dispersed evidence on *Aspergillus* infections in cetaceans and emphasizes its overlooked significance in marine mammal health. While early reports were scarce, particularly before the 2000s [39,42,43,47,49,54,59,60], accumulated findings now indicate that *Aspergillus* spp.—especially *A. fumigatus*—may be a more frequent and impactful pathogen than previously recognized. In fact, the number of published cases has increased steadily since the early 2000s, raising the possibility that the true incidence of aspergillosis in cetaceans is on the rise, and improvements in surveillance and reporting have led to greater detection. More reports were found in tropical and temperate regions, but it is currently unclear whether this higher prevalence is just related to a higher sampling effort in these localities and/or a higher likelihood of infection in tropical-temperate areas (as observed in humans [61]).

Among the *Aspergillus* species identified, *A. fumigatus* was the most frequently reported, accounting for the majority of invasive infections and most of the attributable deaths. This predominance is consistent with its well-characterized virulence factors, including thermotolerance and angioinvasion. Indeed, only a single case of invasive disease attributed to an unidentified *Aspergillus* species has been documented: i.e., a fatal case of otitis media and interna in *P. phocoena* [53]. Other *Aspergillus* species, including *A. niger* [28,50,51], *A. flavus* [60], *A. versicolor* [28,51], *A. tubingensis*, and *A. ustus* [51], have been reported sporadically, primarily in cases of colonization or in localized, non-lethal lesions. Some were identified as co-infecting agents in cutaneous lesions, such as *A. terreus* and *A. tubingensis* in *P. crassidens* [51]. Aside from *A. terreus*, none were convincingly associated with disseminated or fatal infections. However, the clinical relevance of these less common species should be interpreted with caution, since several reports lacked molecular confirmation and relied solely on phenotypic identification, increasing the likelihood of misidentification, particularly within morphologically similar taxa.

Despite the apparent prominence of *A. fumigatus* as a virulent and adaptable pathogen, the clinical significance of non-*fumigatus* isolates remains uncertain but likely limited in most cases. It remains unclear whether these species represent true pathogens in cetaceans or merely reflect colonization, environmental contamination, or post mortem overgrowth. Even for *A. fumigatus*, its role as a primary cause of mortality should be interpreted cautiously. In several cases, invasive aspergillosis was diagnosed in animals with known or suspected immunosuppressive conditions, such as morbillivirus infection or even captivity, hence acting primarily as a compensatory rather than additive cause of mortality. From a conservation perspective, this distinction is important, as it highlights the need to address the primary drivers of immunosuppression and ecological stress that may predispose cetaceans to fungal infections.

Moreover, only a minority of published cases identified the fungal agent beyond the genus level. Many studies reported only the presence of *Aspergillus* spp. or *Aspergillus*-like structures, without species-level identification [17,24,39,41,43,44,45,47,48,49,56,58,59].

Antifungal susceptibility testing was rarely performed, which is a notable concern given the increasing emergence of azole-resistant *A. fumigatus* strains arising from environmental acquisition of resistance [31]. Resistance was confirmed in two studies [32,33], with isolates harbouring specific mutations linked to environmental exposure to azole fungicides such as TR34/L98H in wild *P. phocoena* individuals in the Netherlands [33]. These findings highlight a still-neglected connection between anthropogenic contamination, marine fungal epidemiology, and marine mammal pathology. In this context, cetaceans may serve not only as vulnerable hosts but also as inadvertent sentinels of antifungal resistance dissemination in aquatic ecosystems.

*Tursiops truncatus* and *P. phocoena* were the most frequently affected species and, interestingly, different infectious tropism was noted, with otic infections being more frequent in porpoises. While this may reflect species susceptibility, it likely also results from their greater availability for study, either due to their prevalence in captivity or because they commonly strand along coasts accessible to researchers. Large offshore or pelagic species are underrepresented [25,38], likely not due to lower susceptibility but rather to the reduced likelihood of carcass recovery in a diagnostically suitable condition. In line with cases of invasive aspergillosis in terrestrial animals, including humans [62], most cases involved respiratory tract disease, particularly of the lower airways, with invasive features often noted post mortem. In several reports, pathology extended beyond the airways, with evidence of angioinvasion, central nervous system involvement, or myocarditis [24,25,47]. Particularly, tracheitis and bronchial occlusion were also frequent [9,17], suggesting that upper airway obstruction may contribute to stranding or respiratory failure. Central nervous system involvement was reported in multiple cases [37,42,45,55], raising concern about the neurotropic capabilities of *Aspergillus* in immunocompromised or co-infected individuals. Interestingly, an apparent tropism of *Aspergillus* spp. for the auditory system was noted in *P. phocoena*, a feature not observed in other cetacean species. Otitis media and interna were reported in multiple cases [45,53,57], often in association with concurrent meningoencephalitis or pulmonary lesions. The recurrent involvement of the otic region, often marked by suppurative or granulomatous inflammation and occasional erosion of middle ear structures, may indicate anatomical or physiological predispositions unique to this species. These factors warrant further investigation, including a possible association with parasitic infestations such as *S. minor*, which commonly affects the middle and inner ear in this host species, and might serve as a predisposing factor for fungal superinfections [57]. Importantly, such infections could impair echolocation, potentially increasing the risk of disorientation and reduced ability to feed, bycatch, and stranding [57]. The absence of similar findings in other cetaceans, despite numerous reported cases of aspergillosis, underscores a potentially species-specific vulnerability that should prompt further pathological and ecological investigation. In contrast, a subset of reports documented only colonization [44,50,60], particularly in animals under human care, possibly due to easier sampling access.

Co-infections emerged as a frequent and clinically relevant feature in *Aspergillus*-associated disease among cetaceans, reported in almost 60% of the studies analysed. Notably, morbillivirus was the most commonly co-detected agent, described in cases involving harbour porpoises, striped dolphins, and bottlenose dolphins [9,42,45], often in association with disseminated or neurologically invasive fungal infections. Bacterial pathogens (e.g., *P. aeruginosa*, *P. damselae*) were also frequently reported [8,17,38], potentially amplifying respiratory or systemic damage. In some cases, helminths and protozoons, such as *Sarcocystis speeri* or *Stenurus minor*, among others, were found in affected tissues [57], raising the possibility that local tissue disruption facilitated fungal entry. Immunosuppression appears central to the pathogenesis of invasive aspergillosis in cetaceans, often driven by concurrent viral infections such as morbillivirus and exacerbated by pollutant exposure. Viral-induced immune dysfunction likely facilitates fungal invasion and dissemination, as evidenced in dolphins and porpoises affected by both pathogens [8,42,47]. This immunological vulnerability may be further intensified by environmental immunotoxins like organochlorines, which impair host defences and modulate disease severity [9]. In small or stressed populations, such as those recovering from viral outbreaks, secondary fungal infections can act as critical mortality amplifiers [58], emphasizing the need to consider co-infection dynamics and ecosystem health in conservation strategies.

Some reports on animals under human care provide some insights into therapeutic approaches against aspergillosis in cetaceans. Among the few animals treated, outcomes were variable and largely dependent on the clinical context and drug regimen. Resolution was achieved in only four cases treated with systemic antifungals such as itraconazole [54,60], a combination of voriconazole and posaconazole [32], or a multimodal regimen including voriconazole, micafungin, and inhaled amphotericin B, with pharmacologically induced leukopenia by micafungin [52]. In contrast, fluconazole monotherapy [24] was ineffective, due to the intrinsic resistance of *A. fumigatus*. The limited therapeutic success underscores the challenges of antifungal treatment in marine mammals, including late or incorrect diagnosis, difficult infectious source control, drug pharmacokinetics, and the advanced stage at presentation.

Despite increasing documentation of *Aspergillus* infections in cetaceans, conservation implications remain insufficiently addressed in most studies. This is notable given the potential for fungal pathogens to interact with viral coinfections, pollutant exposure, and habitat degradation, compounding their impact on vulnerable populations. A limited number of reports highlighted relevant concerns: in *T. truncatus* and *S. frontalis*, aspergillosis was identified as a potential threat to free-ranging populations [8,26], while studies on harbour porpoises emphasized the association between immunosuppression, pollutant burden, and fungal susceptibility [45,53,57]. In several cases, fungal infections were described in small or isolated populations [37,38,58], raising concern over their potential contribution to localized declines. Some studies also emphasized the need for post mortem surveillance and systematic pathology as tools to detect emerging mycotic diseases and assess broader ecosystem health [38,55]. Furthermore, evidence of environmental antifungal resistance [33] and seasonal fungal fluctuations [28] suggests that anthropogenic pressures may influence both exposure and pathogen dynamics.

However, existing literature is limited by reliance on case reports, uneven geographic and taxonomic coverage, and variable diagnostic standards. Data from captive or stranded individuals may not accurately reflect the health status of free-ranging populations, given their different environmental exposures and potential for post mortem contamination. These limitations underscore the value of developing non-invasive tools—such as environmental DNA and aerial respiratory sampling—to improve surveillance in wild populations. Nonetheless, by consolidating the available literature, this review provides a clearer picture of where knowledge gaps remain. Future efforts should aim for better surveillance, improved diagnostics, and more integrated frameworks that connect wildlife health with conservation and public health concerns. They should aim for standardized and comprehensive reporting. Key recommendations include consistently specifying the year and geographic location of cases; providing detailed methodological descriptions, particularly regarding fungal identification techniques and antifungal susceptibility testing; and clearly distinguishing between colonization and invasive infection. Whenever possible, authors should quantify infection frequency within study populations and describe associated clinical outcomes. Improved reporting of co-infections, environmental context, and potential conservation implications is also encouraged to facilitate comparative analyses and inform integrated One Health strategies. Such efforts will enhance data quality, support epidemiological synthesis, and help bridge current knowledge gaps in marine fungal disease ecology.

## 5. Conclusions

*Aspergillus* spp., particularly *A. fumigatus*, are increasingly recognized as significant pathogens in cetaceans, primarily affecting the respiratory tract but also involving the central nervous and auditory systems, with clinical manifestations showing certain species-tropism. Most studies have been published after 2000 and are predominantly case reports, which reflects a growing awareness but also a lack of systematic surveillance and standardized investigation. Co-infections and environmental stressors, such as pollutants and antifungal resistance, are expected to increase with climate change and likely amplify disease severity. Species identification and antifungal susceptibility testing are inconsistently applied, limiting epidemiological and therapeutic insight. The emergence of azole-resistant strains and its overlap with anthropogenic pressures point to broader ecological and conservation concerns. The potential for *Aspergillus* to influence stranding, morbidity, and even conservation outcomes in vulnerable populations highlights the need for enhanced surveillance, species-specific pathology studies, and integrated One Health strategies.

## Figures and Tables

**Table 1:** Overview of published reports on *Aspergillus* spp. in cetaceans.

Cetacean Species	Type of Study	Number of Animals	Status	Year	Location	Reference
Killer whale (*Orcinus orca* [Linnaeus, 1758])	Case report	1	Captive	NA	Nagoya (Japan)	Abdo et al. [24]
Atlantic spotted dolphin (*Stenella frontalis* [Cuvier, 1829])	Case report	1	Free-living	2020	Florida (USA)	Balik et al. [26]
Northern bottlenose whale (*Hyperodoon ampullatus* [Forster, 1770])	Case report	1	Free-living	2006	North Kessock (Scotland)	Barley et al. [37], Dagleish et al. [38]
Bottlenose dolphin (*Tursiops truncatus* Montagu, 1821)	Case report	1	Captive	NA	Nagoya (Japan)	Bunskoek et al. [32]
*T. truncatus*	Case report	1	Free-living	2012	Florida (USA)	Cassle et al. [8]
*T. truncatus*	Case report	1	Captive	NA	Nagoya (Japan)	Carroll et al. [39]
Harbour porpoise (*Phocoena Phocoena* [Linnaeus, 1758])	Case report	1	Free-living	2006	Arrochar (Scotland)	Dagleish et al. [40]
*T. truncatus*	Case series	4	Captive	1998–2008	USA	Delaney et al. [17]
*T. truncatus*	Original experimental study	32	Captive	1991–2016	USA	Desoubeaux et al. [41]
Stripped dolphin (*Stenella coeruleaoalba* [Meyen, 1833])	Observational epidemiological study	3	Free-living	1990	Catalonia (Spain)	Domingo et al. [42]
*S. coeruleaoalba*	Case report	1	Free-living	2016	Alassio (Italy)	Grattarola et al. [9]
*S. frontalis*, Bryde’s whale (*Balaenoptera edeni* Anderson, 1879)	Case series	2	Free-living	2014, 2016	Santa Catarina (Brazil)	Groch et al. [25]
*T. truncatus*	Case report	1	Free-living	2019	Florida (USA)	Hamel et al. [27]
Northern right whale dolphin (*Lissodelphis borealis* Peale, 1848), *T. truncatus,* Commerson’s dolphin (*Cephalorhynchus commersonii* [Lacépède, 1804])	Case series	3	Free-living	NA	Santa Barbara Channel (USA), Magellan Strait (Chile)	Joseph et al. [43]
Beluga whale (*Delphinapterus leucas* [Pallas, 1776]	Technical note	5	Captive	2021–2023	Nagoya (Japan)	Kamio et al. [44]
*P. phocoena*	Observational epidemiological study	11	Free-living	2012–2016	Netherlands	Kapetanou et al. [45]
Indo-Pacific bottlenose dolphins (*Tursiops aduncus* [Ehrenberg, 1832])	Observational epidemiological study	2	Free-living	2013	(Australia)	Kemper et al. [46]
*O. orca*	Environmental surveillance study	0	Captive	2012–2013	Nagoya (Japan)	Kohata et al. [28]
*T. truncatus*	Case report	1	Free-living	1993	Florida (USA)	Lipscomb et al. [47]
Guiana dolphin (*Sotalia guianensis* [van Bénéden, 1864)	Observational epidemiological study	1	Free-living	2016–2018	Parana (Brazil)	Marutani et al. [48]
*T. truncatus*	Case series	3	Captive	NA	California, Florida (USA)	Migaki & Jones [49]
*T. truncatus*	Observational microbiological study	3	Free-living	2003–2005	South Carolina, Florida (USA)	Morris et al. [50]
False killer whale (*Pseudorca crassidens* Owen, 1846)	Observational ecological and microbiological study	6	Free-living	2009	Cape Peninsula (South Africa)	Mouton et al. [51]
*T. truncatus*	Case report	1	Captive	2019	Nagoya (Japan)	Ohno et al. [52]
*P. phocoena*	Case report	1	Free-living	2001	Kent (England)	Prahl et al. [53]
*T. truncatus*	Case report	1	Captive	1995	California (USA)	Reidarson et al. [54]
*P. phocoena*	Case report	1	Free-living	2021	Schleswig-Holstein (Germany)	Rohner et al. [55]
Franciscana dolphin (*Pontoporia blainvillei* [Gervais & d’Orbigny, 1844])	Case report	1	Free-living	2020	Buenos Aires (Argentina)	Romano et al. [56]
*P. phocoena*	Case report	1	Free-living	NA	Denmark	Seibel et al. [57]
*T. aduncus*	Observational epidemiological study	2	Free-living	2009	Western Australia	Stephens et al. [58]
*T. truncatus*, pygmy sperm whale (*Kogia breviceps* [Blainville, 1838]), narwhal (*Monodon monoceros* Linnaeus, 1758)	Observational epidemiological study	3	Free-living	NA	NA	Sweeney et al. [59]
*P. phocoena*	Retrospective observational study	6	Free-living	NA	Netherlands	Van Dijk et al. [33]
*D. leucas*	Retrospective observational and cross-sectional microbiological study	4	Captive	1994–1995	Vancouver (Canada)	Young et al. [60]

NA denotes that the information was not reported in the study. Note: Entries are organized alphabetically by first author, following standard practice in systematic reviews to maintain consistency with the reference list and facilitate cross-referencing.

**Table 2:** *Aspergillus* species in reported cetacean cases: identification methods and antifungal susceptibility.

Cetacean Species	*Aspergillus* Species	Identification Method	Susceptibility	Reference
Killer whale (*Orcinus orca*)	*Aspergillus* spp.	Histopathology	No	Abdo et al. [24]
Atlantic spotted dolphin (*Stenella frontalis*)	*A. fumigatus*	Histopathology, DNA sequencing	No	Balik et al. [26]
Northern bottlenose whale (*Hyperodoon ampullatus*)	*A. fumigatus*	Histopathology, culture	No	Barley et al. [37], Dagleish et al. [38]
Bottlenose dolphin (*Tursiops truncatus*)	*A. fumigatus*	Culture, galactomannan, DNA sequencing	Yes	Bunskoek et al. [32]
*T. truncatus*	*A. fumigatus*	Culture, histopathology, DNA sequencing	No	Cassle et al. [8]
*T. truncatus*	*Aspergillus* spp.	NA	No	Carroll et al. [39]
Harbour porpoise (*Phocoena phocoena*)	*A. fumigatus*	Culture, histopathology, PCR, DNA sequencing	No	Dagleish et al. [40]
*T. truncatus*	*Aspergillus* spp. One *A. fumigatus*	Culture, histopathology, PCR, DNA sequencing	No	Delaney et al. [17]
*T. truncatus*	*Aspergillus* spp.	NA	NA	Desoubeaux et al. [41]
Striped dolphin (*Stenella coeruleaoalba*)	*A. fumigatus*	Histopathology	No	Domingo et al. [42]
*S. coeruleoalba*	*A. fumigatus*	Culture, DNA sequencing	No	Grattarola et al. [9]
*S. frontalis*, Bryde’s whale (*Balaenoptera edeni*)	*A. fumigatus*	Histopathology, PCR, DNA sequencing	No	Groch et al. [25]
*T. truncatus*	*A. fumigatus*	Histopathology, PCR, DNA sequencing	No	Hamel et al. [27]
Northern right whale dolphin (*Lissodelphis borealis*), *T. truncatus*, Commerson’s dolphin (*Cephalorhynchus commersonii*)	*Aspergillus* spp.	Culture, histopathology	No	Joseph et al. [43]
Beluga whale (*Delphinapterus leucas*)	*Aspergillus*-like	Culture	No	Kamio et al. [44]
*P. phocoena*	*A. fumigatus* (10), *Aspergillus* spp. (1)	Culture, histopathology, PCR, DNA sequencing	No	Kapetanou et al. [45]
Indo-Pacific bottlenose dolphins (*Tursiops aduncus*)	*A. fumigatus*	Culture, not further stated	No	Kemper et al. [46]
*O. orca*	*A. fumigatus, A. niger, A. versicolor*	Culture	No	Kohata et al. [28]
*T. truncatus*	*Aspergillus*-like	Histopathology	No	Lipscomb et al. [47]
Guiana dolphin (*Sotalia guianensis*)	*Aspergillus*-like	Histopathology	No	Marutani et al. [48]
*T. truncatus*	*Aspergillus* spp.	Culture	No	Migaki & Jones [49]
*T. truncatus*	*A. fumigatus*, *A. niger*	Culture	No	Morris et al. [50]
False killer whale (*Pseudorca crassidens*)	*A. versicolor*, *A. niger*, *A. ustus*, *A. terreus*, *A. tubingensis*	Culture, DNA sequencing	No	Mouton et al. [51]
*T. truncatus*	*A. fumigatus*	Culture	No	Ohno et al. [52]
*P. phocoena*	*A. terreus*	Culture	No	Prahl et al. [53]
*T. truncatus*	*A. fumigatus*	Culture, serology	No	Reidarson et al. [54]
*P. phocoena*	*A. fumigatus*	Culture, DNA sequencing	No	Rohner et al. [55]
Franciscana dolphin (*Pontoporia blainvillei*)	*Aspergillus* spp.	Histopathology	No	Romano et al. [56]
*P. phocoena*	*A. fumigatus*	Histopathology	No	Seibel et al. [57]
*T. aduncus*	*Aspergillus* spp.	Histopathology	No	Stephens et al. [58]
*T. truncatus*, pygmy sperm whale (*Kogia breviceps*), narwhal (*Monodon monoceros*)	*Aspergillus* spp.	NA	No	Sweeney et al. [59]
*P. phocoena*	*A. fumigatus*	Culture	Yes	Van Dijk et al. [33]
*D. leucas*	*A. fumigatus*, *A. flavus*, *A. niger*	Culture	No	Young et al. [60]

NA denotes that the information was not reported in the study. Note: Entries are organized alphabetically by first author, following standard practice in systematic reviews to maintain consistency with the reference list and facilitate cross-referencing.

**Table 3:** Clinical and pathological features of *Aspergillus* infection and colonization in cetaceans.

Cetacean Species	Type of Infection	Co-infective Agent	Treatment	Outcome	Reference
Killer whale (*Orcinus orca*)	Lung abscesses and multifocal bronchopneumonia	*Mucorales* and Herpesvirus	Fluconazole	Death	Abdo et al. [24]
Atlantic spotted dolphin (*Stenella frontalis*)	Suppurative tracheitis and bronchopneumonia	*Sarcocystis speeri*, *Treponema* sp.	No	Death	Balik et al. [26]
Northern bottlenose whale (*Hyperodoon ampullatus*)	Encephalitis, tracheitis	*Fusobacterium* sp, *Morganella morganii*, *Photobacterium damselae, Eubacterium*	No	Death	Barley et al. [37], Dagleish et al. [38]
Bottlenose dolphin (*Tursiops truncatus*)	Tracheitis, pneumonia	*Vibrio alginolyticus*	Voriconazole, posaconazole	Resolution	Bunskoek et al. [32]
*T. truncatus*	Bronchopneumonia, encephalitis, sinusitis	Morbillivirus	No	Death	Cassle et al. [8]
*T. truncatus*	Pneumonia	No co-infection	NA	NA	Carroll et al. [39]
Harbour porpoise (*Phocoena phocoena*)	Brain granuloma, pulmonary lymphadenitis	*Pseudalius inflexus*	No	Death	Dagleish et al. [40]
*T. truncatus*	Occlusive tracheitis, bronchopneumonia	No co-infection	No	Death	Delaney et al. [17]
*T. truncatus*	Respiratory or disseminated	NA	NA	NA	Desoubeaux et al. [41]
Striped dolphin (*Stenella coeruleaoalba*)	Necrotizing hemorrhagic encephalitis, granulomatous-necrotizing pneumonia, vasculitis	Morbillivirus	No	Death	Domingo et al. [42]
*S. coeruleoalba*	Occlusive tracheobronchitis	Alphaherpesvirus	No	Death	Grattarola et al. [9]
*S. frontalis*, Bryde’s whale (*Balaenoptera edeni*)	Pyogranulomatous, angioinvasive necrotizing bronchopneumonia, orchitis periorchitis, mesenteric lymphadenitisand pyogranulomatous bronchopneumonia	No co-infection	No	Death	Groch et al. [25]
*T. truncatus*	Multifocal meningoencephalitis, multifocal bronchopneumonia	Morbillivirus	No	Death	Hamel et al. [27]
Northern right whale dolphin (*Lissodelphis borealis*), *T. truncatus*, Commerson’s dolphin (*Cephalorhynchus commersonii*)	Pyogranulomatous pneumonia	Atlantic bottlenose dolphin: *Pseudomonas putrefaciens, Streptococcus* sp. group D, and beta hemolytic *Micrococcus* sp. Northern right whale dolphin: *Pseudomonas* sp and *Escherichia coli* Commerson’s dolphin: *E. coli* and *Staphylococcus aureus*	No	Death	Joseph et al. [43]
Beluga whale (*Delphinapterus leucas*)	Respiratory colonization	No co-infection	No	Health	Kamio et al. [44]
Harbour porpoise	Granulomatous pneumonia, meningoencephalitis, otitis media	No co-infection	No	Death	Kapetanou et al. [45]
Indo-Pacific bottlenose dolphins (*Tursiops aduncus*)	Lung infection	Morbillivirus	No	Death	Kemper et al. [46]
Killer whale	NA	NA	NA	NA	Kohata et al. [28]
*T. truncatus*	Necrotizing ulcerative tracheitis, suppurative hemorrhagic pneumonia, necrotizing myocarditis	Morbillivirus	No	Death	Lipscomb et al. [47]
Guiana dolphin (*Sotalia guianensis*)	Pneumonia	Morbillivirus	No	Death	Marutani et al. [48]
*T. truncatus*	Granulomatous pneumonia, tracheitis	No co-infection	No	NA	Migaki & Jones [49]
*T. truncatus*	Respiratory colonization	No co-infection	No	Health	Morris et al. [50]
False killer whale *(Pseudorca crassidens)*	Skin colonization or infection	Co-colonization between *Aspergillus* species	No	NA	Mouton et al. [51]
*T. truncatus*	Pneumonia	No	Micafungin, amphotericin B	Resolution	Ohno et al. [52]
*P. phocoena*	Otitis media	No	No	Death	Prahl et al. [53]
*T. truncatus*	Pneumonia	*Morganella morganii, Staphylococcus intermedius, Vibrio alginolyticus*	Itraconazole	Resolution	Reidarson et al. [54]
*P. phocoena*	Pyogranulomatous necrotizing pneumonia, lymphadenopathy, purulent, necrotizing meningoencephalitis	*Stenurus minor, Torynurus convolutus, Campula oblonga*	No	Death	Rohner et al. [55]
*Franciscana dolphin (Pontoporia blainvillei)*	Necrotizing pneumonia	*No*	No	Death	Romano et al. [56]
*P. phocoena*	Otitis media	*S. minor*	No	Death	Seibel et al. [57]
*T. aduncus*	Pyogranulomatous bronchopneumonia and necrotizing meningoencephalitis	Morbillivirus	No	Death	Stephens et al. [58]
*P. phocoena*	Pneumonia	NA	No	Death	Van Dijk et al. [33]
*D. leucas*	Respiratory colonization	No	No	Health	Young et al. [60]

Note: Entries are organized alphabetically by first author, following standard practice in systematic reviews to maintain consistency with the reference list and facilitate cross-referencing.

## Data Availability

Databases created for this review are available on request to the authors.

## Supplementary Materials

The following supporting information can be downloaded at https://www.mdpi.com/article/10.3390/biology14060664/s1, PRISMA 2020 Checklist.
